# The bacillus Calmette–Guérin vaccination allows the innate immune system to provide protection from severe COVID-19 infection

**DOI:** 10.1073/pnas.2015234117

**Published:** 2020-09-29

**Authors:** Vincenzo Patella, Gabriele Delfino, Dario Bruzzese, Ada Giuliano, Alessandro Sanduzzi

**Affiliations:** ^a^Division of Allergy and Clinical Immunology, Department of Medicine, Azienda Sanitaria Locale Salerno, “Santa Maria della Speranza” Hospital, 84091 Salerno, Italy;; ^b^Postgraduate Program in Allergy and Clinical Immunology, University of Naples Federico II, 80138 Naples, Italy;; ^c^Department of Public Health, University of Naples Federico II, 80138 Naples, Italy;; ^d^Laboratory of Toxicology Analysis, Department for the Treatment of Addictions, Azienda Sanitaria Locale Salerno, 84124 Salerno, Italy;; ^e^Department of Clinical Medicine and Surgery, Section of Respiratory Disease, University of Naples Federico II, 80138 Naples, Italy;; ^f^Health Education and Sustainable Development, United Nations Educational, Scientific and Cultural Organization, University Federico II, 80138 Naples, Italy

**Fig. 1.**
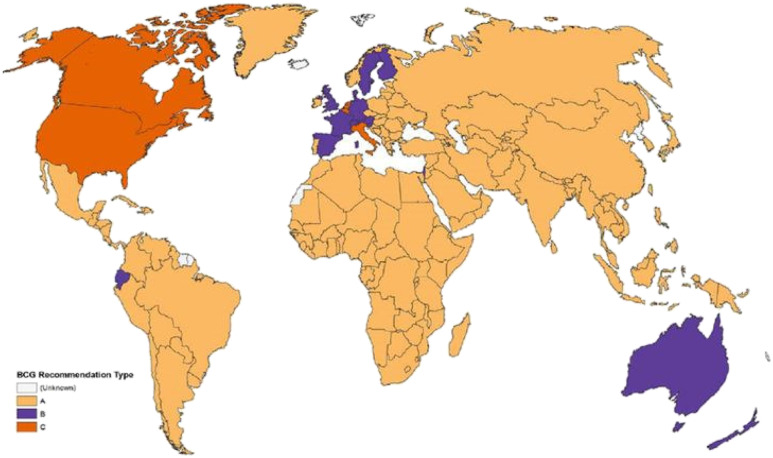
Map displaying bacillus Calmette–Guérin vaccination policy by country. (*A*) The country currently has universal bacillus Calmette–Guérin vaccination program. (*B*) The country used to recommend bacillus Calmette–Guérin vaccination for everyone but currently does not. (*C*) The country never had universal bacillus Calmette–Guérin vaccination programs. Reprinted from ref. (4), which is licensed under CC BY 4.0.

We have read the paper by Escobar et al. (1), and we also support a similar hypothesis (2), which arose from the observation of the so-called Iberian Peninsula paradox. As they also report, in the same geographical area with similar socioeconomic conditions, completely different and particularly high mortality rates have been observed in Spain where tuberculosis vaccination is not mandatory, unlike Portugal (3–5) (Fig.1)

Recently, we conducted a study on Italian physicians, which was subjected to bacillus Calmette–Guérin vaccination, and, in parallel, the doctors in the current context are the subjects most exposed to infection. We identified two groups, “vaccinated” and “unvaccinated,” and verified that there was no significant difference in the incidence of COVID-19 between the two groups (Table 1).

**Table 1.** Demographic characteristics and result of significance between vaccinated and unvaccinated groups of doctors with bacillus Calmette–Guérin during COVID-19 infection

Sex	Participants	Mean age, y	Vaccinated		Not vaccinated	Infected and vaccinated	Infected but not vaccinated	Significance
Females	838 (43.97%)	45.9	480 (45.20%)	358 (42.42%)		—	—	—
Males	1,068 (56.03%)	54.4	582 (54.80%)	486 (57.58%)		—	—	—
Total	1,906	50.7	1,062 (55.72%)	844 (44.28%)		23 (2.17%)	14 (1.66%)	NS (*P* = 0.505)

Would the authors explain the discrepancy between our results and theirs?

1) In our study, we considered the ability of the bacillus Calmette–Guérin vaccine to prevent COVID-19, compared to their work in which the reduction in mortality was assessed. So, the ability of bacillus Calmette–Guérin was examined at different stages of the disease. The trained immunity requires the involvement of innate immunity cells at the early immune response stages; this would ensure that the infectious process is blocked in the bud, without developing clinical symptoms. For this reason, it seemed more appropriate to study the hypothetical bacillus Calmette–Guérin protection from COVID-19.

2) The main problem in studies comparing mortality rates between different countries is the different abilities of national systems to report epidemiological data. In those countries with more precarious welfare systems and greater population density, where the COVID-19 epidemic presented a multilevel emergency (health, social, and political), not all deaths were adequately assessed for the cause. It is therefore probable that several COVID-19 deaths could have not been counted and therefore not reported, resulting in an underestimation of mortality rate. This phenomenon is likely to be more frequent in poorer countries with a lower Human Development Index (HDI). Probably this could partly explain why lower mortality rates have been observed in countries with lower HDI. What happened in China is also significant: The local authorities revised upward the data on the number of deaths. The explanation of high mortality in France and the United Kingdom is not very convincing, despite the bacillus Calmette–Guérin vaccination; the authors justify the ineffectiveness of the trained immunity with the age of administration of bacillus Calmette–Guérin vaccine (in many cases, it is not carried out during early childhood, but in the second and third stages of childhood). According to mechanisms of trained immunity, it is conceivable that this can be sustained even in the older child, where there is an innate immune system preponderance compared to adaptive immunity that is still incomplete. This is also motivated by regression of time, which does not begin before puberty.

Furthermore, the indiscriminate use of bacillus Calmette–Guérin vaccine could present a serious risk of depletion of the stocks currently available globally, with the consequent lack of a preventive measure versus tuberculosis. As pointed out by the authors, the recommendations of the World Health Organization (6) reiterate that it is necessary to await the results of ongoing clinical trials (7, 8).
